# Supplementary material for: Pyruvate Dehydrogenase Kinase 1 inhibition mediated oxidative phosphorylation enhancement in cartilage promotes osteoarthritis progression
Source: BMC Musculoskelet Disord. 2023 Jul 20;24:597. doi: 10.1186/s12891-023-06585-6 (PMC10357736; doi:10.1186/s12891-023-06585-6)
Supplement: Supplementary file 1 — Additional file 1. [file 12891_2023_6585_MOESM1_ESM.zip › KEGG.Gsea.1653622432464/KEGG_CYTOSOLIC_DNA_SENSING_PATHWAY.html]

Details for gene set KEGG\_CYTOSOLIC\_DNA\_SENSING\_PATHWAY[GSEA]

|  || Dataset | 55457\_collapsed\_to\_symbols.cls.cls #OA\_versus\_normal.cls.cls #OA\_versus\_normal\_repos |
| Phenotype | cls.cls#OA\_versus\_normal\_repos |
| Upregulated in class | normal |
| GeneSet | KEGG\_CYTOSOLIC\_DNA\_SENSING\_PATHWAY |
| Enrichment Score (ES) | -0.33274633 |
| Normalized Enrichment Score (NES) | -1.2423942 |
| Nominal p-value | 0.16129032 |
| FDR q-value | 0.32243952 |
| FWER p-Value | 1.0 |
Table: GSEA Results Summary

  

Fig 1: Enrichment plot: KEGG\_CYTOSOLIC\_DNA\_SENSING\_PATHWAY      
 Profile of the Running ES Score & Positions of GeneSet Members on the Rank Ordered List

  

| SYMBOL | TITLE | RANK IN GENE LIST | RANK METRIC SCORE | RUNNING ES | CORE ENRICHMENT || 1 | TREX1 | three prime repair exonuclease 1 [Source:HGNC Symbol;Acc:HGNC:12269] | 393 | 0.709 | 0.0152 | No |
| 2 | CASP1 | caspase 1 [Source:HGNC Symbol;Acc:HGNC:1499] | 431 | 0.694 | 0.0571 | No |
| 3 | POLR3K | RNA polymerase III subunit K [Source:HGNC Symbol;Acc:HGNC:14121] | 651 | 0.605 | 0.0791 | No |
| 4 | PYCARD | PYD and CARD domain containing [Source:HGNC Symbol;Acc:HGNC:16608] | 1164 | 0.484 | 0.0705 | No |
| 5 | IFNA8 | interferon alpha 8 [Source:HGNC Symbol;Acc:HGNC:5429] | 1206 | 0.475 | 0.0979 | No |
| 6 | POLR3C | RNA polymerase III subunit C [Source:HGNC Symbol;Acc:HGNC:30076] | 1504 | 0.429 | 0.1025 | No |
| 7 | CXCL10 | C-X-C motif chemokine ligand 10 [Source:HGNC Symbol;Acc:HGNC:10637] | 1709 | 0.396 | 0.1122 | No |
| 8 | POLR3B | RNA polymerase III subunit B [Source:HGNC Symbol;Acc:HGNC:30348] | 2220 | 0.336 | 0.0942 | No |
| 9 | IL18 | interleukin 18 [Source:HGNC Symbol;Acc:HGNC:5986] | 2472 | 0.306 | 0.0944 | No |
| 10 | RELA | "RELA proto-oncogene, NF-kB subunit [Source:HGNC Symbol;Acc:HGNC:9955]" | 3942 | 0.161 | -0.0095 | No |
| 11 | IFNA4 | interferon alpha 4 [Source:HGNC Symbol;Acc:HGNC:5425] | 4282 | 0.129 | -0.0275 | No |
| 12 | IRF7 | interferon regulatory factor 7 [Source:HGNC Symbol;Acc:HGNC:6122] | 4411 | 0.120 | -0.0297 | No |
| 13 | NFKB1 | nuclear factor kappa B subunit 1 [Source:HGNC Symbol;Acc:HGNC:7794] | 4626 | 0.102 | -0.0398 | No |
| 14 | AIM2 | absent in melanoma 2 [Source:HGNC Symbol;Acc:HGNC:357] | 4892 | 0.080 | -0.0552 | No |
| 15 | IFNA2 | interferon alpha 2 [Source:HGNC Symbol;Acc:HGNC:5423] | 5869 | 0.004 | -0.1309 | No |
| 16 | IKBKB | inhibitor of nuclear factor kappa B kinase subunit beta [Source:HGNC Symbol;Acc:HGNC:5960] | 5914 | 0.001 | -0.1342 | No |
| 17 | IFNA21 | interferon alpha 21 [Source:HGNC Symbol;Acc:HGNC:5424] | 6127 | -0.015 | -0.1497 | No |
| 18 | DDX58 | DExD/H-box helicase 58 [Source:HGNC Symbol;Acc:HGNC:19102] | 6480 | -0.041 | -0.1745 | No |
| 19 | CCL5 | C-C motif chemokine ligand 5 [Source:HGNC Symbol;Acc:HGNC:10632] | 6584 | -0.048 | -0.1794 | No |
| 20 | IFNA10 | interferon alpha 10 [Source:HGNC Symbol;Acc:HGNC:5418] | 6682 | -0.054 | -0.1834 | No |
| 21 | IFNA5 | interferon alpha 5 [Source:HGNC Symbol;Acc:HGNC:5426] | 6755 | -0.059 | -0.1852 | No |
| 22 | IFNA6 | interferon alpha 6 [Source:HGNC Symbol;Acc:HGNC:5427] | 7272 | -0.101 | -0.2188 | No |
| 23 | POLR1D | RNA polymerase I and III subunit D [Source:HGNC Symbol;Acc:HGNC:20422] | 7611 | -0.128 | -0.2369 | No |
| 24 | IFNA7 | interferon alpha 7 [Source:HGNC Symbol;Acc:HGNC:5428] | 8533 | -0.196 | -0.2958 | No |
| 25 | IFNA1 | interferon alpha 1 [Source:HGNC Symbol;Acc:HGNC:5417] | 8640 | -0.204 | -0.2910 | No |
| 26 | IL33 | interleukin 33 [Source:HGNC Symbol;Acc:HGNC:16028] | 9078 | -0.238 | -0.3096 | Yes |
| 27 | ADAR | adenosine deaminase RNA specific [Source:HGNC Symbol;Acc:HGNC:225] | 9195 | -0.247 | -0.3027 | Yes |
| 28 | POLR3F | RNA polymerase III subunit F [Source:HGNC Symbol;Acc:HGNC:15763] | 9229 | -0.250 | -0.2891 | Yes |
| 29 | POLR1C | RNA polymerase I and III subunit C [Source:HGNC Symbol;Acc:HGNC:20194] | 9405 | -0.265 | -0.2856 | Yes |
| 30 | IKBKE | inhibitor of nuclear factor kappa B kinase subunit epsilon [Source:HGNC Symbol;Acc:HGNC:14552] | 9646 | -0.287 | -0.2858 | Yes |
| 31 | POLR3G | RNA polymerase III subunit G [Source:HGNC Symbol;Acc:HGNC:30075] | 10018 | -0.323 | -0.2938 | Yes |
| 32 | ZBP1 | Z-DNA binding protein 1 [Source:HGNC Symbol;Acc:HGNC:16176] | 10164 | -0.337 | -0.2833 | Yes |
| 33 | IFNB1 | interferon beta 1 [Source:HGNC Symbol;Acc:HGNC:5434] | 10801 | -0.406 | -0.3066 | Yes |
| 34 | IRF3 | interferon regulatory factor 3 [Source:HGNC Symbol;Acc:HGNC:6118] | 10879 | -0.414 | -0.2859 | Yes |
| 35 | IKBKG | inhibitor of nuclear factor kappa B kinase regulatory subunit gamma [Source:HGNC Symbol;Acc:HGNC:5961] | 10986 | -0.428 | -0.2665 | Yes |
| 36 | CCL4 | C-C motif chemokine ligand 4 [Source:HGNC Symbol;Acc:HGNC:10630] | 11211 | -0.458 | -0.2543 | Yes |
| 37 | NFKBIB | NFKB inhibitor beta [Source:HGNC Symbol;Acc:HGNC:7798] | 11277 | -0.468 | -0.2292 | Yes |
| 38 | TBK1 | TANK binding kinase 1 [Source:HGNC Symbol;Acc:HGNC:11584] | 11326 | -0.474 | -0.2023 | Yes |
| 39 | CHUK | component of inhibitor of nuclear factor kappa B kinase complex [Source:HGNC Symbol;Acc:HGNC:1974] | 11624 | -0.521 | -0.1918 | Yes |
| 40 | RIPK1 | receptor interacting serine/threonine kinase 1 [Source:HGNC Symbol;Acc:HGNC:10019] | 11953 | -0.584 | -0.1797 | Yes |
| 41 | IL1B | interleukin 1 beta [Source:HGNC Symbol;Acc:HGNC:5992] | 12105 | -0.622 | -0.1513 | Yes |
| 42 | IL6 | interleukin 6 [Source:HGNC Symbol;Acc:HGNC:6018] | 12460 | -0.730 | -0.1317 | Yes |
| 43 | POLR3D | RNA polymerase III subunit D [Source:HGNC Symbol;Acc:HGNC:1080] | 12490 | -0.744 | -0.0859 | Yes |
| 44 | MAVS | mitochondrial antiviral signaling protein [Source:HGNC Symbol;Acc:HGNC:29233] | 12646 | -0.822 | -0.0449 | Yes |
| 45 | NFKBIA | NFKB inhibitor alpha [Source:HGNC Symbol;Acc:HGNC:7797] | 12817 | -1.001 | 0.0065 | Yes |
Table: GSEA details [plain text format]

  

Fig 2: KEGG\_CYTOSOLIC\_DNA\_SENSING\_PATHWAY      
 Blue-Pink O' Gram in the Space of the Analyzed GeneSet

  

Fig 3: KEGG\_CYTOSOLIC\_DNA\_SENSING\_PATHWAY: Random ES distribution      
 Gene set null distribution of ES for **KEGG\_CYTOSOLIC\_DNA\_SENSING\_PATHWAY**
